# Direct measurement of the capillary condensation time of a water nanobridge

**DOI:** 10.1038/s41598-018-32021-0

**Published:** 2018-09-14

**Authors:** Miguel V. Vitorino, Arthur Vieira, Carolina A. Marques, Mario S. Rodrigues

**Affiliations:** 1 Biosystems & Integrative Sciences Institute, Faculdade de Ciências, Universidade de Lisboa, 1749-016 Lisboa, Portugal; 2 Departamento de Física, Faculdade de Ciências, Universidade de Lisboa, 1749-016 Lisboa, Portugal

## Abstract

Water menisci wet all sorts of cavities, produce among the most intense forces at the nanoscale and play a role in many physical and chemical processes. The physical properties of these menisci are therefore relevant to understand a multitude of phenomena at the nanoscale where these are involved. Here, using a force feedback microscope, we directly measured the capillary condensation time of a water meniscus, by approaching two surfaces at different speeds and monitoring the relative position of the surfaces at the instant the meniscus is formed.

## Introduction

If one wants to describe the nanoscale with only a few words, one of those can be’wet’. This is because when water vapor is confined below critical dimensions, a water capillary bridge spontaneously condenses^1–3^. It is an ubiquitous phenomenon, present in all sorts of cavities^4^ and produces one of the most intense forces at the nanoscale. This phenomenon directly influences a diversity of systems, including sand castles^5,6^, protein folding^7,8^, insect attachment mechanisms^9^, surface chemistry^3^ or friction^10–12^ and, due to the strong adhesion forces it produces, it also has an impact on micro- and nanoelectromechanical systems^3,13–15^. Nanoscale probing tools such as Atomic Force Microscopy (AFM), suffer directly from the presence of capillary bridges, that often produce jump-to-contact of the tip towards the sample surface, preventing accurate tip-sample force characterization^16^.

Capillary condensation has therefore been the target of a large research effort, particularly since the invention of advanced tools such as the surface force apparatus^17^ or more recently the atomic force microscope^18^. AFM allows for the direct measurement of the interaction between a sharp tip and a sample of interest with nanometer spatial resolution^19,20^. It has played a key role in the investigation of adhesion processes between surfaces^21,22^, the structuring of liquid thin films^23^, the atomic mechanisms of friction^24–26^ and has contributed to the determination of the nanoscale behaviour of capillary bridges^27^, their nanomechanical impedance^28,29^ or rupture kinetics^30,31^. However and despite the extensive amount of research, there are still many unknowns in the behavior of nanocapillary bridges.

Some attention has been given to the nucleation time of these capillaries. Nucleation of the water meniscus has so far been difficult to study with conventional AFM techniques due to the jump-to-contact mechanism that prevents the acquisition of the static tip-sample interaction with soft cantilevers. Thus, some authors have used friction force microscopy to measure the friction force between tip and sample and study its dependence with sliding speed^24,25,32^. While these results and models have allowed for the characterization of the nanobridge formation as a thermally-activated process, taking place in the timescale of the millisecond, these measurements did not probe directly the time involved in the formation of these bridges in a clearly defined local geometry. On the other hand Sung *et al*.^33^ have circumvented AFM limitations by using a quartz tuning fork (QTF)-based AFM to probe the formation of the water meniscus by monitoring in real time the oscillations of the QTF, taking advantage of its high force constant that prevents the mechanical instability present in common AFM. In this way the authors were able to measure the activation time of the nanobridges, together with their nucleation rate. These measurements suggest nucleation times on the order of hundreds of milliseconds to a few seconds. However, since an oscillator with a settling time on the order of 40 ms is used, any dynamics faster than this timescale is not monitored. This prevents the measurement of the phenomena reported in nanofriction experiments (nucleation times on the order of hundreds of microseconds to a few milliseconds) and does not allow to probe the formation of the nanobridge in a conventional AFM force-distance curve (in which the tip is close or in contact with the sample some tens of milliseconds). These conflicting results, thus, give rise to questions regarding the influence of nucleation mechanisms and local geometry on the phenomenon of capillary nucleation.

To address this problem, in this work we present a direct measurement of the capillary condensation time using a custom-made force feedback microscope (FFM). This instrument allows the measurement of the complete interaction curve by avoiding the jump-to-contact^16^ mechanism. Since it uses common AFM cantilevers, we are thus capable of probing this nucleation in a clearly defined local geometry with a suitable timescale.

## Experimental approach

We studied the formation of a meniscus formed between a carbon spherical tip and a cleaved mica surface. The capillary force arising in this system was measured with FFM, which allows the measurement of the complete tip-sample interaction. This is achieved using a feedback loop that counterbalances the tip-sample force by displacing the cantilever anchoring point (see details in Methods section). This instrument has reproducibly showcased its ability to stabilize a capillary bridge^16,29,34^.

The experiment relies on two basic assumptions: first, there is a critical tip-sample distance, *D*_*c*_, that favors the condensation of the capillary bridge and second, within the range of velocities used, neither the critical distance nor the condensation time depend on the tip-sample approach velocity, *v*_*ts*_. This critical distance *D*_*c*_ depends on the meniscus equilibrium curvature, usually assumed to be given by the Laplace-Kelvin equation:

1 $$\frac{{\gamma }_{lv}}{{r}_{K}}={\rho }_{l}{k}_{B}Tln({\varphi }^{-1})$$

where *γ*_*lv*_ is the liquid-vapor surface tension, *r*_*K*_ the Kelvin radius and *ρ*_*l*_, *k*_*B*_, *T* and *ϕ* are the density, Boltzmann constant, temperature and relative humidity, respectively. The left-hand side of the equation represents the Laplace pressure. The distance that favors nucleation has been studied by several authors^3,35,36^ and can be written as:

2 $${D}_{c}=g{r}_{k}+{z}_{0}$$

where *z*_0_ is a constant that depends on the definition of the tip-sample distance. Here, *z* = 0 is defined as the position at which the force is null, corresponding to the minimum of the energy, which is in fact the value we can determine with more accuracy. Thus, *z*_0_ corresponds to the indentation such that contact repulsive forces cancel out adhesion forces. For a pure water meniscus forming between two flat surfaces with water contact angle *θ*, *g* = 2cos*θ*. In practice, surfaces are not perfectly flat and factors such as the geometry of the system^37^, the presence of adsorbed liquid films on the surfaces^3^ or the presence of nonvolatile solutes in the meniscus^38^ have been shown to influence the nucleation critical distance. Since all measurements are performed at the same experimental conditions, we assume *D*_*c*_ remains constant in our measurements. We note that its *a priori* knowledge is not required for the analysis that follows.

At tip-sample distances smaller than *D*_*c*_ the liquid phase is energetically favorable, leading to condensation which occurs within a certain mean time *t*_*c*_, which we call condensation time. If the tip approaches the sample at a speed *v*_*ts*_ it will travel a certain distance, while the condensation process is ongoing, from the onset of nucleation, at *D*_*c*_, until the position where the meniscus is detected, *D*_*d*_. This position corresponds to the instant when a liquid bridge connects tip and sample and is associated with an abrupt and significant vertical jump in the tip-sample force. This traveled distance is, on average, *v*_*ts*_*t*_*c*_. The tip-sample distance where the meniscus is detected, *D*_*d*_, will thus be dependent on the approach speed, the critical distance and the condensation time through the relation:

3 $$\overline{{D}_{d}}={D}_{c}-{v}_{ts}\overline{{t}_{c}}$$

Note that we do not directly measure the critical distance, since it corresponds to the case when the approach speed tends to zero.

**Figure 1:**
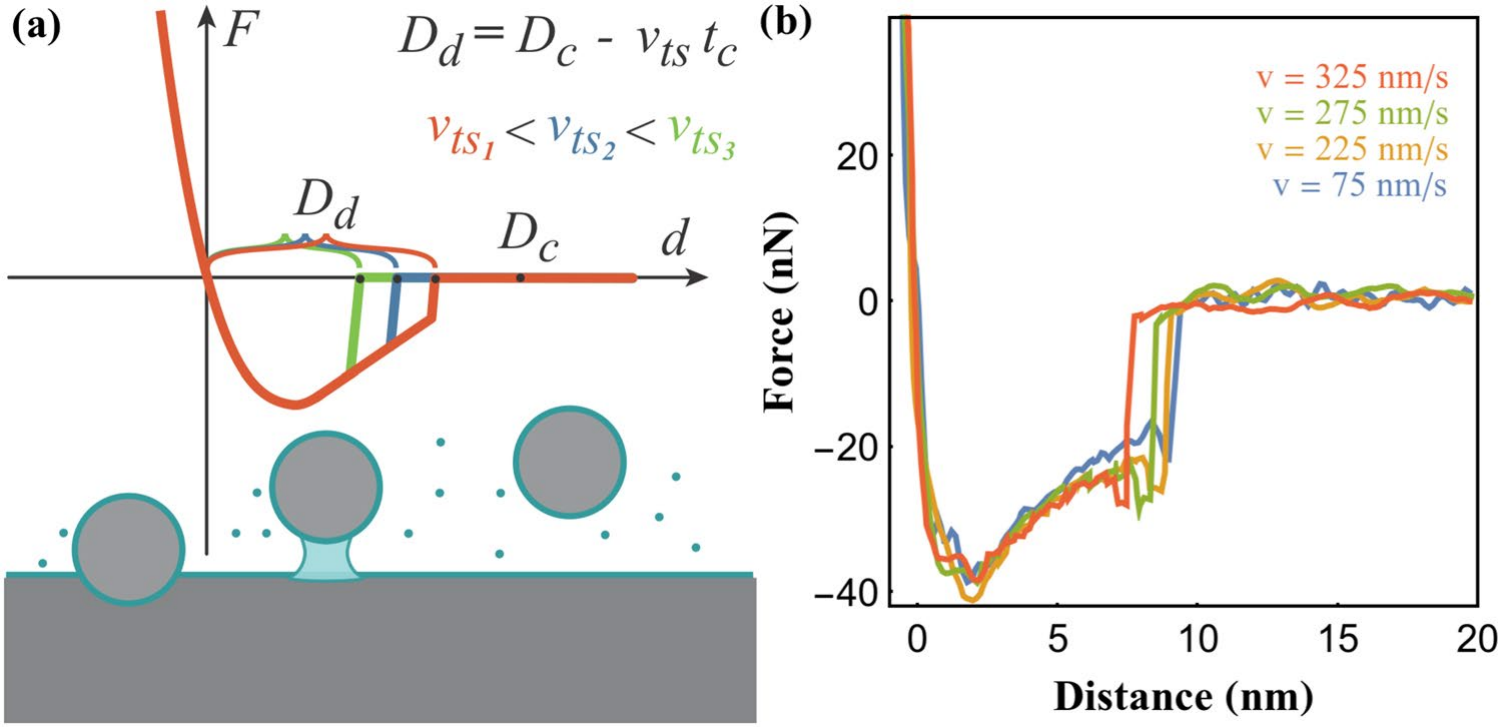
(**a**) Schematics of the experimental approach developed in this work. If the nucleation critical distance and the condensation time are approximately constant within a small range of approach speeds, we expect the meniscus to be formed at progressively smaller tip-sample distances with increasing tip-sample approach speed; (**b**) force distance curves at different approach speeds acquired with the FFM. The instant when the meniscus is formed is characterized by a vertical jump in the force. Note that there is a slight overshoot characteristic of feedback systems, that, however, does not compromise our measurement.

If *v*_*ts*_*t*_*c*_ is sufficiently small, then *D*_*d*_ ≈ *D*_*c*_ and we can assume that the mean condensation time is approximately constant, i.e. *t*_*c*_(*D*_*d*_) ≈ *t*_*c*_(*D*_*c*_). In a force-distance curve measured with the FFM the formation of the meniscus is detected by identifying the abrupt jump in the tip-sample force. The fact that the tip does not jump to contact means that it is possible to measure accurately the distance from this point to the sample. This setup provides a method for probing both the critical distance and the condensation time of the nanobridge, by monitoring the meniscus condensation distance while changing the approach speed. We expect a linear relation between the distance at which the meniscus is detected *D*_*d*_, that gets progressively smaller with increasing approach speed, and speed as represented in Fig. 1(a).

## Results and Discussion

Figure 1(b) presents several force-distance curves performed at different approach speeds with the force feedback microscope. These curves exhibit a sudden and attractive jump, that we associate with the meniscus formation, *D*_*d*_. At this position there is a slight overshoot of the force which is natural to the feedback loop response. Before this jump there is no significant tip-sample force detected and after it an approximately linear trend can be observed, followed by the sharp increase in force when tip and sample are in mechanical contact. This approximately linear trend with the distance is expected for the evolution of capillary forces between the surfaces involved^3^ and is routinely observed in the study of capillary interactions^3,27,28,39^.

**Figure 2:**
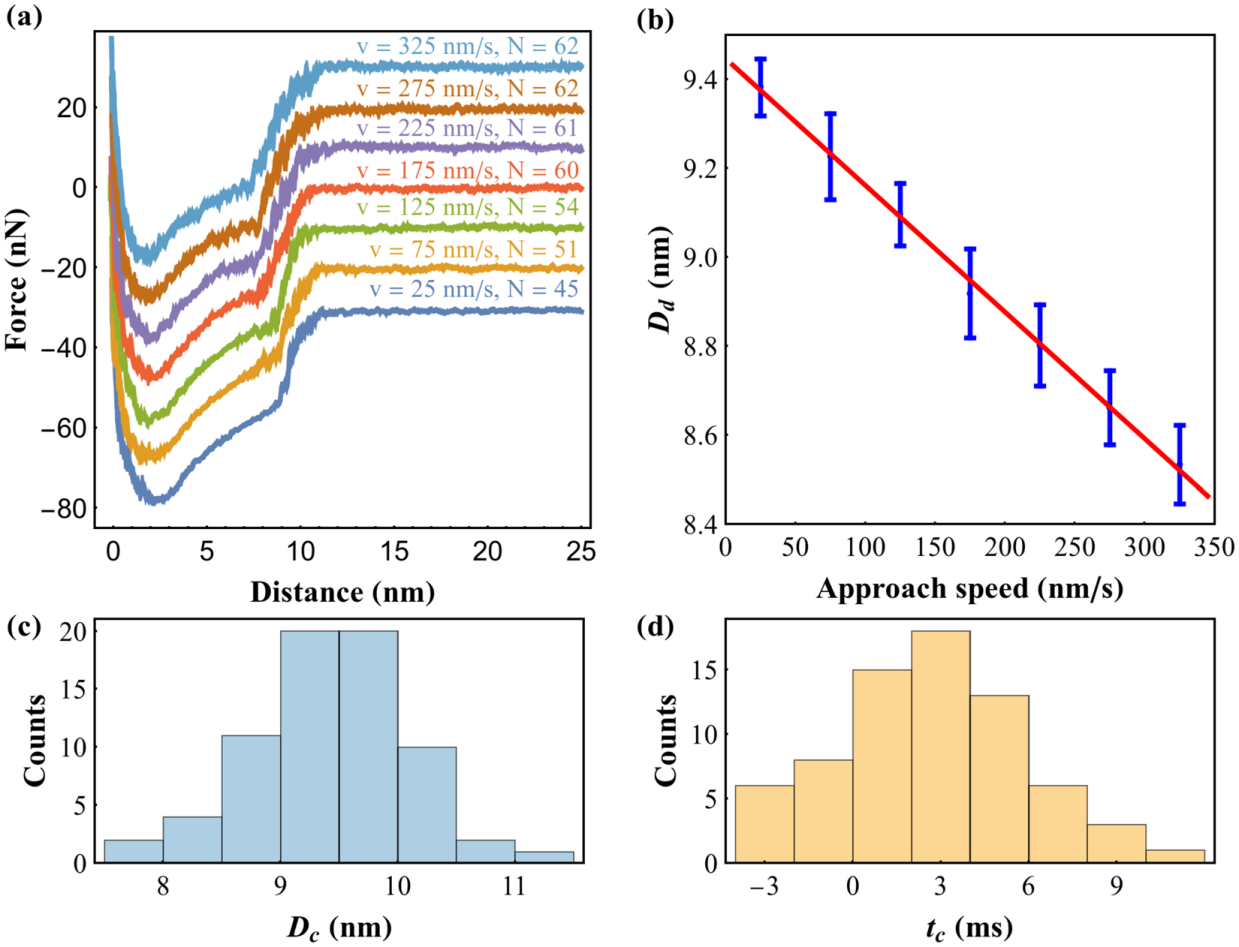
Capillary bridges probed with Force Feedback Microscopy: (**a**) average force-distance curve acquired at each speed, shifted vertically for clarity. A clear reduction of the capillary nucleation distance is observed. Approach velocity and number of curves averaged are written above each curve; (**b**) average detected nucleation distance dependence on the tip-sample approach speed. A linear relation between the two was found. Error bars represent the standard deviation of the mean of the nucleation distance for each speed; The analysis of each series of force-distance curves yields individual values of *D*_*c*_ and *t*_*c*_, which results in histograms of the critical nucleation distance (**c**) and the condensation time (**d**).

Because capillary condensation is a stochastic process^32^, it is necessary to repeat this experiment several times, to obtain the average condensation time and critical distance for this system. Figure 2 presents the experimental results obtained in one such experiment. We repeated 70 times a series of force-distance curves that swept 7 different approach speeds in the range of 25–325 *nm*/*s* and aligned them all according to the tip-sample contact position (for details see Methods section). The force-distance curves were grouped by their different approach speeds. The average curve for each approach speed can be observed in Fig. 2(a). While the superposition of all the curves makes it difficult to distinguish the average position of the force jump for the different speeds, the comparison of the linear portion of the force curves clearly indicates a decrease of the detected condensation distance with approach speed, as expected. Additionally Fig. 2(b) presents the average of the *D*_*d*_ obtained from each individual force-distance curve for each approach speed. The error bars represent the standard error of the distributions. It is possible to verify the linear behavior predicted in Eq. (3). The linear fit to this data yields *D*_*c*_ = (9.45 ± 0.03) nm and *t*_*c*_ = (2.8 ± 0.2) ms. The stochastic nature of the data can be better seen on the histograms also plotted in Fig. 2. For each velocity sweep acquired we have performed a linear fit and obtained its own *D*_*c*_ and *t*_*c*_. Figure 2(c,d) presents the histograms of these values. The corresponding average values *D*_*c*_ = (9.47 ± 0.02) nm and *t*_*c*_ = (2.81 ± 0.06) ms reproduce quite well the previous results.

Our results confirm the previously observed relatively long timescales that appear to govern capillary bridge dynamics^10,32,33,40^. It is noteworthy to compare these results with the ones obtained in nanofriction experiments by Szoszkiewicz *et al*.^32^ at room temperature. While these authors measured nucleation times of about 4 ms for temperatures similar to the ones employed in the present study, they have characterized this nucleation in a much less humid atmosphere. Therefore, since the mean curvature radius of the nanomeniscus in our experiments is at least one order of magnitude larger than the one assumed there, both results cannot be directly juxtaposed. However it is clear from our results that the condensation of water menisci occurs at timescales on the order of a few ms for a simple sphere-plane geometry.

**Figure 3:**
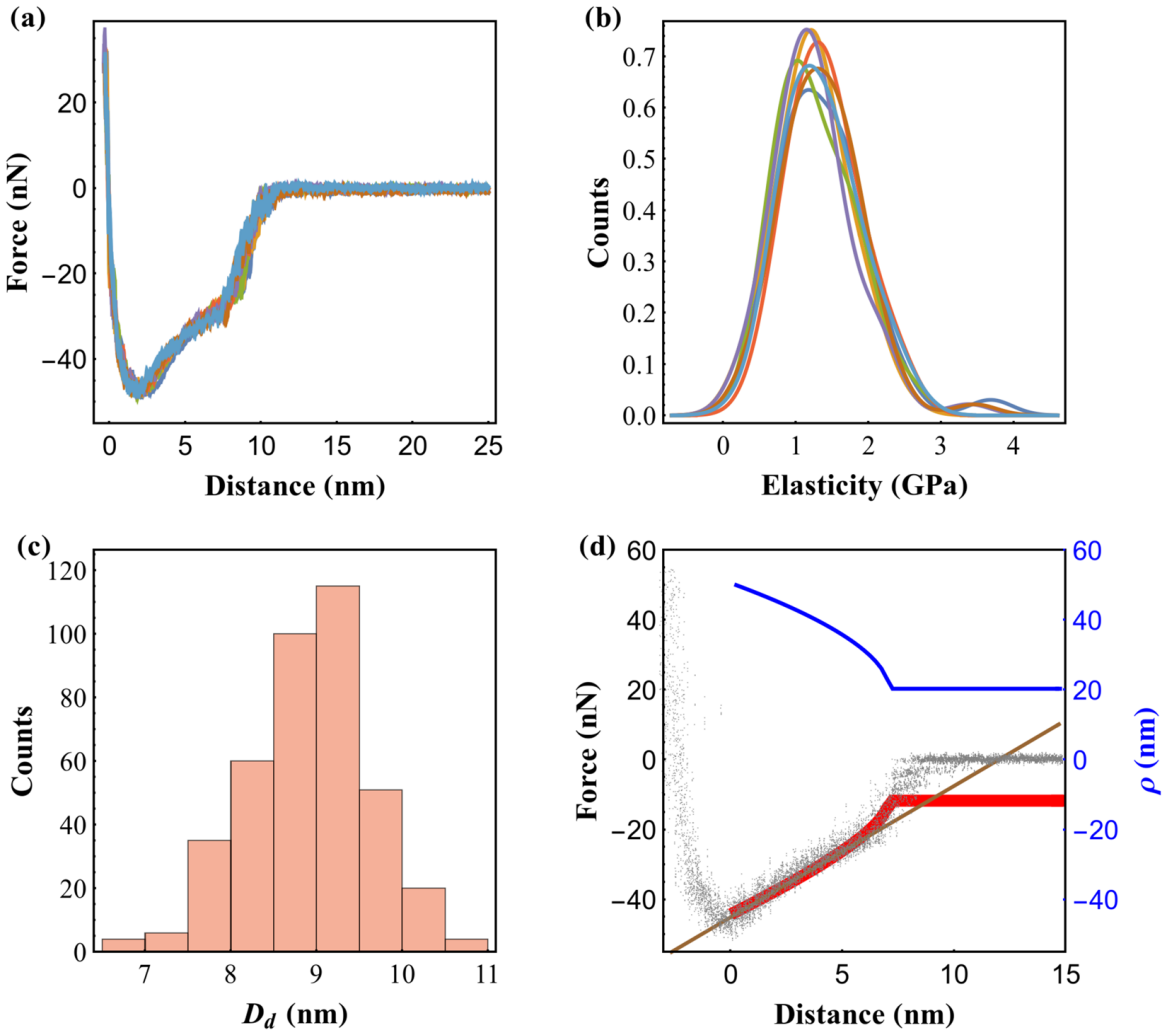
Quality control of the experiment: (**a**) average of the force-distance curves with the same speed, exhibiting a very reasonable alignment of the repulsive part of the interaction. (**b**) histogram of the elasticity obtained from the hertzian fit to the individual force-distance curves, grouped into series corresponding to each approach velocity, showing no dependence on approach speed; (**c**) histogram of the nucleation distance obtained from all the individual force-distance curves; (**d**) Calculated force curve using a spherical approximation (red), linear trend (brown, see text for details) and cross sectional radius (blue), the values at distances greater than 7 nm have no physical meaning. Black dots correspond to the force-distance curve performed at a tip-sample approach speed of 25 nm/s.

The observed critical nucleation distances, *D*_*c*_, of ~9.5 nm, or ~7 nm if we consider the onset of contact at the minimum of the force-distance curves, are not compatible with Eq. (1) if one uses water bulk values and vapor pressure measured few cm from the sample surface. We have measured thousands of capillary bridges at the same relative humidity and consistently measure about 7 nm. This is a very reproducible value. For a sphere-plane geometry and taking into account that the surfaces are not both hydrophilic, our measurement of *D*_*c*_ is at least 4 times greater than what is predicted by Eq. (1), even considering the pre-existence of water layers at the surfaces prior to the nucleation of the meniscus. The apparent disparity between Laplace-Kelvin theory and nanoscale measurements is routinely observed by other groups^39–42^ and several reasons have been pointed out. To our knowledge, reasonable agreement between nanoscale measurements and Eq. (1) has only been found for very high humidity using aggressive cleaning procedures for sample and probe^43^. We note that at 60% relative humidity the expected critical distance for flat perfectly wetting surfaces is ~2 nm, while the interparticle distance and mean free path of the water molecules are substantially greater. It is therefore reasonable to believe that Eq. (1) may not apply directly to water menisci at this humidity or that some effective parameter must be considered, such as effective vapor pressure at vanishing distances from the surface. It is not the goal of the present work to enter in elaborate arguments relative to the critical distance of such capillary bridges. However, for the sake of completeness, we show in Fig. 3(d) an ensemble of force curves measured at the lowest speed and the calculation of the force produced by the meniscus using a spherical approximation. The force was computed as *F* = − *πρ*^2^*γ*/*r*_*k*_ − 2*πργ* where the first term is the Laplace pressure and the second term is the force exerted by the contact line, with *ρ* the cross sectional radius of the meniscus parallel to the surface. We assumed a contact angle of 0° and 90° between the liquid and mica surface and carbon tip respectively. To adjust the expression above we used *γ* = 0.06 J/m^2^ and *r*_*k*_ = 15 nm for the liquid surface tension and Kelvin radius respectively. The result for *ρ* for the spherical approximation is also shown. We have imposed equilibrium by stating 1/*r*_*k*_ = 1/*ρ* + 1/*r* where *r* is the meniscus curvature perpendicular to the surface. Additionally, we also plot the linear expression $F=-\,4\pi \gamma R(\cos \,\theta -z/2{r}_{k})$^3^. Since two different surfaces are involved, evaluating cos*θ* requires the combination of the wetting angles between the liquid and the two surfaces. Adjusting this expression to our data has yielded a value of 0.4 for this parameter.

If one assumes smaller wetting angle between the liquid and the spherical tip, this has the effect of decreasing the Kelvin radius, while maintaining critical distance but simultaneously increasing the magnitude of the force, hence, to adjust the data that would require to lower the surface tension which is about 0.83 of the expected value of 72 mJ/m^2^. Note that a correction factor on the cantilever stiffness of about 1.2 would be sufficient to correct this. We have used the thermal method for calibrating the cantilever, such error is likely to occur. Because the critical distance is larger than what Eq. (1) predicts it is perhaps worthwhile to estimate its volume. From Fig. 3(d) we estimate *ρ* = 21 nm just when the meniscus forms. Using 7 nm for the meniscus height, this gives a volume of about 10 × 10^3^ nm^3^, or about 4 × 10^5^ water molecules.

In conclusion, using Force Feedback Microscopy we have directly measured the meniscus critical nucleation distance and condensation time. The geometry of our system can clearly be described as a nanomeniscus formed between a spherical tip and a flat sample and our meniscus is formed without any contact between sample and tip. We believe that this very simple experiment can provide additional insight to this discussion and contribute to a better understanding of the underlying mechanisms that govern the formation of water nanobridges.

## Methods

### Force Feedback Microscopy

FFM circumvents some of the limitations imposed in conventional AFM^16,44^. It relies on the inclusion of an extra feedback loop controlling the position (relative to the laboratory reference frame) of the free end of the cantilever at all times. This position is measured with a fiber-optic interferometer^45^, which differs from the more usual laser beam deflection system where, instead of the position, the angle of the cantilever free end is measured. Control of the tip position is performed by adjusting the position of the clamped end of the cantilever, with an additional piezoelectric actuator. This, in turn, exerts a force on the tip, that counterbalances the tip-sample force, leaving the tip position unchanged. The feedback loop is implemented through a proportional-integral-derivative controller. The controller proportional gain can therefore be thought as an artificial stiffness, that will add to the cantilever. This extra force gradient stabilizes the tip position, allowing us to avoid the jump to contact mechanism common in conventional AFM. In an FFM set up the interaction force, *F*_*i*_ is obtained by measuring the displacement of the cantilever base needed to keep the tip at constant position: *F*_*i*_ = *x*_*b*_*k*_*l*_, with *x*_*b*_ the cantilever base motion and *k*_*l*_ the cantilever spring constant.

FFM has been used in the past to characterize the dynamical properties of water bridges^29^, as well as to study such different themes as cell mechanics^46^ or nanofriction^47^. Furthermore, the idea of using a feedback loop to avoid the jump to contact mechanism has already been addressed in different measurement schemes^48^ and is particularly suitable for the measurement of capillary interactions at the nanoscale^39,41^, providing important insights only possible by measuring the complete interaction curve between tip and sample.

### Experimental Setup

We used a custom-made force feedback microscope. The time constant of the FFM feedback loop was measured prior to the experiments and adjusted with the feedback gains^49^ to be below 1 ms. The AFM cantilever used for these experiments was the B150_FMR cantilever from *Nanotools* featuring high density carbon tips with nominal radius of 150 nm. Its stiffness was determined to be 2.8 N/m, using the Thermal method^50^. The resonance frequency of the lever was 85.8 kHz and the Q-factor 150, giving a settling time on the order of 550 *μ*s, low enough to probe dynamics on the order of the ms. The sample was freshly cleaved muscovite mica. The experiments took place in ambient temperature, 298 K and relative humidity, 60%, conditions. To reduce time-dependent effects such as humidity changes or drifts in the position of the tip, a custom-made software ensured a cyclic operation of the sample scanner at different speeds, repeating series of force-distance curves with ascending or descending approach velocities. The interferometric detector of the microscope allowed for the accurate calibration of several of its subsystems, including the force detector and scanner displacements. These showed no significant dependence on the approach speed. The approach speed was scanned between 25 nm/s and 325 nm/s.

### Data Processing

FFM relies on the control of the tip position, *x*_*t*_, measured with interferometric detection, that constitutes the feedback error. Overall Δ*x*_*t*_ ≪ Δ*x*_*b*_. However, the error contribution slightly affects the slope of the contact part. For this reason, all force curves were corrected with their respective feedback error yielding a very good match of the contact part for all curves as can be seen in Fig. 3(a). The measurement of *D*_*d*_, the distance from the meniscus formation point to the tip-sample contact, requires the precise determination of the tip-sample contact point. To ensure this, we have fitted the contact part of each curve with the Hertz Contact Model^51^, allowing us to obtain the reduced Young’s Modulus of tip and sample for each velocity. Figure 3(b) presents the histogram for this elasticity parameter obtained for every force-distance curve performed, grouped into series corresponding to each approach velocity. It can be seen that these do not exhibit any correlation with the approach speed. We measured an average elasticity of 1.3 GPa. While the elasticity of mica is expected to be significantly higher^52^, this value is reasonably justified considering that the stiffness of the employed cantilever is relatively low, which affects the measurement of the Young’s modulus of the sample^20^. Moreover, the goal of this measurement is not to accurately obtain the elasticity of mica, but to show that the reference position is robustly determined regardless of speed. The position at which the meniscus forms is determined by finding the minimum of the numerical derivative of the force curve. Because not all force curves have sufficient signal to noise ratio to accurately detect the position at which nucleation occurs and/or the exact contact position, we have automatically rejected curves for which the determined *D*_*d*_ is outside a window of 5 nm centered around the mean. Figure 3(c) shows an histogram of *D*_*d*_ containing all the curves that are inside this window and were used in this study. We have obtained 70 repetitions of 7 speeds in a total of 490 curves. After this rejection, 395 curves were analyzed.

## Data Availability

The datasets generated during and/or analyzed during the current study are available from the corresponding author on reasonable request.
